# Comparison of respiratory and skin disorders between residents living close to and far from Solous landfill site in Lagos State, Nigeria

**DOI:** 10.4102/phcfm.v13i1.2677

**Published:** 2021-04-30

**Authors:** Abdul-Hakeem O. Abiola, Folahanmi C. Fakolade, Babatunde A. Akodu, Adebola A. Adejimi, Oluwagbemiga A. Oyeleye, Ganiyu A. Sodamade, Aisha T. Abdulkareem

**Affiliations:** 1 Department of Community Health and Primary Care, College of Medicine, University of Lagos, Lagos, Nigeria; 2 Department of Engineering, Faculty of Civil and Environmental Engineering, University of Lagos, Lagos, Nigeria

**Keywords:** Lagos, landfill sites, Nigeria, respiratory disorders, skin disorders, solid waste

## Abstract

**Background:**

Solid waste dump sites have proven to have potentially high risk to human health as it serves as a source of air, soil and underground water pollution.

**Aim:**

This study aimed to assess and compare the knowledge, respiratory disorders and skin disorders between residents living close to and far from landfill sites in Lagos State, Nigeria.

**Setting:**

Igando (a community within 5 km close to) and Badagry (a community beyond 5 km from) Solous Landfill sites in Lagos state, Nigeria.

**Methods:**

A comparative cross-sectional study amongst 103 respondents recruited from each of the two study sites by multistage sampling method was carried out. Data were collected using pretested, structured, interviewer-administered questionnaire, and analysed using Microsoft Excel 2007, EPI Info 7 and WinPepi statistical software packages. Student *t*-test, Fisher’s exact and Chi-square tests were carried out. The *p* ≤ 0.05 was considered statistically significant.

**Results:**

The mean age of Igando and Badagry respondents was 34.18 ± 10.21 years and 32.62 ± 9.84 years, respectively. The two communities differed significantly (*p <* 0.0001) with respect to distance of workplace from landfill site and duration of stay in the residential location. The mean knowledge score of respondents on respiratory and skin disorders associated with solid waste dump site close to landfill sites (82.53 ± 20.60) was statistically significantly higher than those of respondents far from landfill sites (71.84 ± 20.57) (*p* = 0.0003). Respiratory and skin disorders experiences of respondents close to landfill sites were statistically significantly (*p* < 0.0001) higher than those of residents far from landfill sites with respect to wheezing, frequent sneezing, unpleasant odour, fever and skin rashes.

**Conclusion:**

Respiratory and skin disorders experienced by respondents close to landfill sites are higher than those of residents far from landfill sites. Landfill sites should not be located close to human settlements.

## Introduction

Solid waste consists of items discarded every day, which are no longer valuable.^1^ Solid waste is of major concern today in urban areas because of progressive increase in the population in these areas, subsequently resulting in the increase of volume of waste generated.^1^ These wastes include food wastes, dead animals, yard wastes, containers, clothes, furniture and other inorganic materials that are considered as wastes by humans.^2^ They are either in solid or semi-solid form and can also be classified as bio-degradable or non-degradable amongst other classifications.^2,3^

Because waste generation by humans is inevitable, the problem of waste is often not whether or not it is generated but how it is managed after it is being generated.^2^ Failure to manage it will result in human communities being subjected to unpleasant odour, possible methane explosions, emergence of disease vectors and rodents who feed on the wastes and unwholesome living environments.^3^

However, because of the attendant problems of disposing off these wastes, especially in urban areas where there are numerous demands on land space, urban communities are constantly evolving ways of managing the wastes generated by the teeming populations.^4^ Examples of some of the waste management strategies adopted include landfill, incineration, disposal in water bodies, disposal to open land space.^4^ However, in both developed and developing nations, land filling is the cheapest, simplest and most cost-effective way of disposing solid wastes.^4^

Waste disposal sites pose two chronic problems for communities.^5^ Firstly, they are an aesthetic nuisance that impacts negatively on the environment.^5^ Secondly, more vexatious problem is created by disease-carrying agents that transmit bacteria from landfills to nearby human populations.^5^

The Health Protection Agency revealed that long-term exposure to air pollutants from waste increases the risk of mortality, especially from cardiovascular diseases and lung cancer.^6^ Short-term exposure causes cardio-respiratory diseases, including an increase in deaths from heart attacks and respiratory diseases.^7^ Some of the air pollutants include: particles, polychlorinated dibenzo-p-dioxins and polychlorinated dibenzo-p-furans (commonly called dioxins) and other carcinogens such as the polycyclic aromatic hydrocarbons.^8^

Proper management of solid waste is a major concern, especially in the urban areas, where the fast growing population is not under check and as such human settlements are as close to landfills, incinerators and open-waste sites.^8^ Solid waste dump sites have also been proven to have potentially higher generic risk of causing respiratory diseases to human health because it serves as a source of airborne pollution.^8^ The Health Protection Agency emphasises that if wastes are properly managed through modern municipal waste incinerators, only a very small contribution to local concentration of air pollutants would emanate from it.^9^ Thus, any potential damage to the health of those living close-by is likely to be very small, if detectable.^9^ This study was therefore carried out to assess and compare the knowledge and experience of respiratory and skin disorders between residents living near (Igando) and far away from (Badagry) solid waste dump site in Lagos state, Nigeria.

## Methods

### Description of study area

Lagos State is a state in the south-western geopolitical zone of Nigeria. Lagos State is bounded on the north and east by Ogun State. In the west, it shares boundaries with the Republic of Benin. Behind its southern borders lies the Atlantic Ocean. Lagos State is divided into five administrative divisions, which are further divided into 20 Local Government Areas (LGAs). Sixteen of the LGAs comprise the Metropolitan Lagos whilst four LGAs (Badagry, Ikorodu, Ibeju-Lekki and Epe) are the suburban part of Lagos.

Alimosho LGA is the largest LGA in Lagos state, with 1 288 714 inhabitants, according to the official 2006 Census. It is subdivided into six LCDAs, namely Igando/Ikotun LCDA (study site), Agbado/Oke-odo LCDA, Ayobo/Ipaja LCDA, Alimosho LG, Egbe/Idimu LCDA and Mosan Okunola LCDA.

Badagry LGA is a coastal town with a population of 241 093 inhabitants according to the official 2006 Census. It is subdivided into three Local Council Development Areas (LCDA), namely Olorunda LCDA, Badagry LCDA and Badagry west LCDA.

Whilst the state is essentially a Yoruba-speaking environment, it is a socio-cultural melting point attracting both Nigerians and foreigners alike. Lagos State Waste Management Authority (LAWMA) is the agency responsible for the collection and disposal of municipal and industrial waste, as well as for the provision of commercial waste services to the State and Local Governments.^10^ Dumpsite Management is a unit under Waste Management Services provided by LAWMA. There are three major landfill sites (Solous, Olushosun and Abule-Egba landfill sites) and two temporary sites serving Lagos State. Solous landfill sites are located in Igando.

The Solous Sites (Solous I, II and III) are situated along Lagos State University – Iba Road. Soluos I has been inactive since 2006. Soluos II and III are on 7.8 ha and 5 ha of land, respectively, with each having an average lifespan of five years. Each of the sites receives an average of 2250 m^3^ of waste per day.

### Study design and sample size calculation

The study design was a comparative cross-sectional study of the respiratory and skin disorders between residents living near and residents living far away from Solous landfill sites, in Lagos State. The minimum sample size of 103 per group was obtained using the formula for two independent study groups and response rate of 100%:

$$n=\frac{2{\left(Z_{\alpha }+Z_{\beta }\right)}^{2}pq}{d^{2}}$$ [Eqn 1]

where *n* = the desired sample size per group; Z_α_ = the standard normal deviation corresponding to 95% confidence interval = 1.96; Z_β_ = the standard normal deviation corresponding to 80% statistical power = 0.842; *p* = (*p*_1_ + *p*_2_)/2; *p*_1_ = prevalence of adverse health effect amongst those who live close to solid waste dump site from a previous study = 0.23^5^; *p*_2_ = prevalence of adverse health effect amongst those who live away from solid waste dump site from a previous study = 0.15^5^; *q* = 1 − *p* and *d* = *p*_2_−*p*_1_.

The sampling units were the household heads. The respondents were selected by multi-stage sampling method: In the first stage, Alimosho LGA was purposely selected because it is close to Solous landfill sites. Badagry LGA was also purposely selected because it is far away from Solous landfill sites. In the second stage, one political ward (Igando/Egan ward) out the 13 political wards in Alimosho LGA was purposively selected because it is close to (less than 200 m from) Solous landfill sites and one political ward (Ibereko ward) out of the 10 political wards in Badagry LGA was selected by simple random method using balloting procedure. In the third stage, one residential area (Lanre residential area) out of the 25 residential areas in Igando/Egan ward and one residential area (Low-cost housing estate) out of the 17 residential areas in Ibereko ward were selected by a simple random sampling method using balloting procedure. In the fourth stage, 103 households out of the 1000 household units in the Lanre residential area, Igando and 103 households out of the 600 household units in Low-cost housing estate in Ibereko were selected by systematic sampling method with a sampling interval of 6. The list of households in Lanre housing estate and the list of households in Low-cost housing estate, Ibereko, served as the sampling frames. The household heads of each selected household were interviewed.

### Data collection method

Data were collected from the respondents using a pretested, semi-structured interviewer-administered questionnaire. The questionnaire was divided into three parts, Section A: Socio-demographic characteristics of the respondents such as age, sex, tribe, level of education, employment status; Section B: Knowledge about health problems that may arise as a result of living close to solid waste dump site and Section C: Self-reported respiratory and skin disorders the respondents had experienced in the last 6 months.

### Data analysis

Data were analysed using Microsoft Excel 2007, EPI Info 7 and WinPepi statistical software packages. Each correct response to the knowledge question was scored one mark whilst incorrect or non-response was scored zero. The total score obtained by each respondent was converted to percentage and graded as good (≥ 50%) and poor (< 50%). The mean ± standard deviation (SD) knowledge score was computed for each group. Frequency distributions and cross tabulations to examine relationships between variables were carried out. Chi-square and Fisher’s exact tests were used to compare differences between proportions whilst student *t*-test was used for comparison of means. *p* ≤ 0.05 was considered statistically significant. Data collation and analysis were carried out in 2018.

### Ethical considerations

Ethical approval to conduct the study was received from the Health Research and Ethics Committee of the Lagos University Teaching Hospital – HREC/VOLXVI/APP/624.

A consent form was issued to each participant and information about the participants was treated confidential. A participant had the right to decline from participating at any stage of the study.

## Results

All 103 questionnaires administered to each group were retrieved and analysed giving a response rate of 100%.

### Socio-demographic characteristics

The mean age of the Igando respondents was 34.18 ± 10.21 years, whilst that of Badagry was 32.62 ± 9.84 years. Majority of the respondents were in the age group of 21–40 years. There were equal number of males and females in Igando whilst in Badagry, the male respondents were more and they made up 58.3% of the Badagry respondents. Majority of the respondents were married in Igando (55.3%) and single in Badagry (54.4%). Forty-six (44.4%) and 47 (45.6%) of the respondents in Igando and Badagry, respectively, had tertiary education. The workplace of 79.6% and 15.5% of Igando and Badagry respondents, respectively, were near the dumpsite. The mean ± SD of length of stay in residential location was 5.76 ± 3.59 years and 14.29 ± 9.70 years for Igando and Badagry respondents, respectively. Igando and Badagry respondents differed significantly with respect to the distance of workplace from the landfill site (*p* < 0.0001) and length of stay in residential location (*p* < 0.0001) (Table 1).

**TABLE 1:** Socio-demographic characteristics of respondents.

Socio-demographic characteristics	Close to landfill site (Igando) *n* = 103			Far from landfill site (Badagry) *n* = 103			Statistics			
	*n*	%	Mean ± SD	*n*	%	Mean ± SD	*ᵪ*^2^	*t*	*df*	*p*
**Age (years)**										
≤ 20	1	1.1	-	7	6.7	-	-	-	-	-
21–40	84	81.6	-	81	78.6	-	-	-	-	-
41–60	13	12.7	-	12	11.7	-	-	-	-	-
61–80	5	4.6	-	3	2.9	-	-	-	-	-
Mean ± SD	-	-	34.18 ± 10.21	-	-	32.62 ± 9.84	-	1.12	204	0.27
**Gender**										
Male	52	50.5	-	60	58.3	-	-	-	-	-
Female	51	49.5	-	43	41.7	-	0.96	-	1	0.33
**Marital status**										
Single	39	37.9	-	56	54.4	-	-	-	-	-
Married	57	55.3	-	45	43.6	-	-	-	-	-
Divorced	0	0.0	-	1	1.0	-	-	-	-	-
Widowed	7	6.8	-	1	1.0	-	-	-	-	0.014*
**Educational status**										
No formal education	1	1.0	-	5	4.9	-	-	-	-	-
Primary	8	8.1	-	11	10.7	-	-	-	-	-
Secondary	48	46.5	-	40	38.8	-	-	-	-	-
Tertiary	46	44.4	-	47	45.6	-	6.27	-	3	0.106
**Workplace near dumpsite?**										
Yes	82	79.6	-	16	15.5	-	-	-	-	-
No	21	20.4	-	87	84.5	-	82.23	-	1	< 0.0001
**Duration of stay in residential location (years)**										
0–10	93	90.0	-	44	42.7	-	-	-	-	-
11–20	9	9.0	-	36	35.0	-	-	-	-	-
21–30	1	1.0	-	18	17.5	-	-	-	-	-
31–40	0	0.0	-	5	4.8	-	-	-	-	-
Mean ± SD	-	-	5.76 ± 3.59	-	-	14.29 ± 9.70	-	8.37	204	< 0.0001

*df*, degree of freedom; SD, standard deviation; *t*, t-test.

* , Fisher’s exact.

### Knowledge on health effects of living close to landfill site

Knowledge of health-related conditions associated with solid waste dump site was generally good in the two study groups except in the aspect that respiratory infections can be caused by living near a solid waste dump site. Only 41.75% of Igando respondents reported that residents could contract respiratory infection by living near solid waste dump site, whilst 30.10% of Badagry respondents agreed that residents could contract respiratory infection by living near solid waste dump sites (Table 2). The mean knowledge score of respondents close to landfill sites (82.53 ± 20.60) was statistically significantly (*p* = 0.0003) higher than those of respondents far away from landfill site (71.84 ± 20.57). Majority of respondents in Igando (98.1%) and Badagry (97.1%) had good knowledge of respiratory and skin disorders associated with solid waste dump site (Table 3). There was no statistically significant relationship recorded between any of the socio-demographic characteristics and the level of knowledge expressed by Igando and Badagry respondents (Table 4).

**TABLE 2:** Knowledge of respondents on skin and respiratory conditions associated with solid waste dump site.

Knowledge of skin and respiratory conditions	Correct response Study area			
	Close to landfill site (Igando) (*n* = 103) Frequency		Far from landfill site (Badagry) (*n* = 103) Frequency	
	*n*	%	*n*	%
Solid waste dump sites near residential area have a negative impact on the health of residents	96	93.2	75	72.8
Respiratory infections can be caused by living near a solid waste dump site	43	41.8	31	30.1
Fever because of respiratory infection can be contracted by living near a solid waste dump site	93	90.3	69	67.0
Living near solid waste dump sites can cause skin rashes	70	68.0	75	72.8
Solid waste dump sites can pollute the air	102	99.0	97	94.2
Solid waste dump sites can cause infestation of rats	92	89.3	82	79.6
Solid waste dump sites can cause infestation of flies	99	96.1	89	86.4

**TABLE 3a:** Score and grade of knowledge of respondents on respiratory and skin disorders associated with solid waste dump site.

Score of knowledge	Study area		Statistics		
	Close to landfill site (Igando) (*n* = 103) Mean ± SD	Far from landfill site (Badagry) (*n* = 103) Mean ± SD	*t*	*df*	*p*
**Knowledge score**					
Mean knowledge score (%)	82.53 ± 20.60	71.84 ± 20.57	3.73	204	0.0003

*df*, degree of freedom; SD, standard deviation; *t, t*-test.

**TABLE 3b:** Score and grade of knowledge of respondents on respiratory and skin disorders associated with solid waste dump site.

Grade of knowledge	Close to landfill site (Igando) (*n* = 103) Frequency		Far from landfill site (Badagry) (*n* = 103) Frequency		Statistics and *p*
	*n*	%	*n*	%	
**Knowledge grade**					
Good	101	98.1	100	97.1	1.00
Poor	2	1.9	3	2.9	-

* , Fisher’s exact.

**TABLE 4:** Effects of socio-demographic characteristics on the level of knowledge of respondents.

Socio-demographic characteristics	Near dump site (Igando) (*n* = 103)						Statistics			Far from dump site (Badagry) (*n* = 103)						Statistics		
	Good			Poor			*t*	*df*	*p*	Good			Poor			*t*	*df*	*p*
	*n*	%	Mean ± SD	*n*	%	Mean ± SD				*n*	%	Mean ± SD	*n*	%	Mean ± SD			
**Age (years)**																		
≤ 20	1	1.0	-	0	0.0	-	-	-	-	22	22.0	-	0	0.0	-	-	-	-
21–30	41	40.2	-	1	100	-	-	-	-	43	43.0	-	1	33.3	-	-	-	-
31–40	29	28.4	-	0	0.0	-	-	-	-	19	19.0	-	2	66.7	-	-	-	-
41–50	9	8.8	-	0	0.0	-	-	-	-	9	9.0	-	0	0.0	-	-	-	-
51–60	2	2.0	-	0	0.0	-	-	-	-	2	2.0	-	0	0.0	-	-	-	-
61–70	4	3.9	-	0	0.0	-	-	-	-	0	0.0	-	0	0.0	-	-	-	-
Not given	16	15.7	-	0	0.0	-	-	-	-	5	5.0	-	0	0.0	-	-	-	-
Total	102	100	-	1	100	-	-	-	-	100	100	-	3	100	-	-	-	-
Mean ± SD	-	-	34.24 ± 10.26	-	-	29.00 ± 0.00	0.51	85	0.61	-	-	28.46 ± 9.89	-	-	33.67 ± 7.77	0.90	96	0.37
**Gender**																		
Male	51	50.0	-	0	0.0	-	-	-	-	58	58.0	-	2	66.7	-	-	-	-
Female	50	49.0	-	1	100	-	-	-	-	42	42.0	-	1	33.3	-	-	-	-
Not given	1	1.0	-	0	0.0	-	-	-	-	0	0.0	-	0	0.0	-	-	-	-
Total	102	100	-	1	100	-	-	-	1.00*	100	100	-	3	100	-	-	-	1.000*
**Educational status**																		
No formal education	1	1.0	-	0	0.0	-	-	-	-	5	5.0	-	0	0.0	-	-	-	-
Primary	8	7.8	-	0	0.0	-	-	-	-	9	9.0	-	1	33.3	-	-	-	-
Secondary	45	44.1	-	1	100	-	-	-	-	36	36.0	-	0	0.0	-	-	-	-
Tertiary	44	43.1	-	0	0.0	-	-	-	-	40	40.0	-	2	66.7	-	-	-	-
Others	0	0.0	-	0	0.0	-	-	-	-	9	9.0	-	0	0.0	-	-	-	-
Not given	4	3.9	-	0	0.0	-	-	-	-	1	1.0	-	0	0.0	-	-	-	-
Total	102	100	-	1	100	-	-	-	1.00*	100	100	-	3	100	-	-	-	0.4478*
**Workplace near dumpsite?**																		
Yes	81	79.4	-	1	100	-	-	-	-	16	16.0	-	0	0.0	-	-	-	-
No	21	20.6	-	0	0.0	-	-	-	-	84	84.0	-	3	100	-	-	-	-
Total	102	100	-	1	100	-	-	-	1.00*	100	100	-	3	100	-	-	-	1.00*
**Duration of stay in residential location (years)**																		
0–5	53	52.0	-	0	0.0	-	-	-		18	18.0	-	1	33.3	-	-	-	-
6–10	36	35.3	-	1	100	-	-	-		20	20.0	-	0	0.0	-	-	-	-
11–15	9	8.8	-	0	0.0	-	-	-		19	19.0	-	1	33.3	-	-	-	-
16–20	1	1.0	-	0	0.0	-	-	-		12	12.0	-	0	0.0	-	-	-	-
21–25	0	0.0	-	0	0.0	-	-	-		6	6.0	-	0	0.0	-	-	-	-
26–30	0	0.0	-	0	0.0	-	-	-		10	10.0	-	0	0.0	-	-	-	-
31–35	0	0.0	-	0	0.0	-	-	-		1	1.0	-	0	0.0	-	-	-	-
36–40	0	0.0	-	0	0.0	-	-	-		4	4.0	-	0	0.0	-	-	-	-
Not given	3	2.9	-	0	0.0	-	-	-		10	10.0	-	1	33.3	-	-	-	-
Total	102	100	-	1	100	-	-	-		100	100	-	3	100	-	-	-	-
Mean ± SD	-	-	5.75 ± 3.61	-	-	7.00 ± 0.00	0.35	98	0.73	-	-	14.43 ± 9.73	-	-	8.00 ± 7.07	0.93	90	0.36
**Marital status**																		
Single	39	38.2		0	0.0	-	-	-	-	55	55.0	-	1	33.3	-	-	-	-
Married	56	54.9		1	100	-	-	-	-	44	44.0	-	1	33.3	-	-	-	-
Divorced	0	0.0		0	0.0	-	-	-	-	0	0.0	-	1	33.3	-	-	-	-
Widowed	7	6.9		0	0.0	-	-	-	-	1	1.0	-	0	0.0	-	-	-	-
Total	102	100		1	100	-	-	-	1.00*	100	100	-	3	100	-	-	-	0.06*

*df*, degree of freedom; SD, standard deviation; *t, t*-test.

* , Fischer’s exact.

### Respiratory and skin disorders

Respondents from Igando reported the experience of the respiratory and skin disorders associated with living close to the solid waste dump site more than those who live in Badagry, except common cold which was higher amongst Badagry respondents than Igando respondents. In Igando, 80.6% of the respondents experience frequent sneezing whilst only 48.5% of the Badagry respondents had similar experience. The same pattern was reported for odours, 87.4% was recorded in Igando, whilst 47.6% was recorded in Badagry (Table 5).

**TABLE 5:** Respiratory and skin disorders experienced by respondents in the last 6 months.

Respiratory and skin disorders	Close to landfill site (Igando) (*n* = 103)		Far from landfill site (Badagry) (*n* = 103)		Statistics		
	*n*	%	*n*	%	*ᵪ*^2^	*df*	*p*
Common cold	46	44.7	52	50.5	0.49	1	0.49
Wheezing	76	73.8	33	32.0	34.37	1	< 0.0001
Frequent sneezing	83	80.6	50	48.5	21.73	1	< 0.0001
Cough	72	69.9	58	56.3	3.52	1	0.06
Odours	90	87.4	49	47.6	3.52	1	< 0.0001
Fever	82	79.6	56	54.4	13.72	1	< 0.0001
Skin rashes	67	65.1	24	23.3	34.72	1	< 0.0001

*df*, degree of freedom.

Bivariate statistical analysis revealed that reported respiratory and skin disorders of respondents close to the landfill sites were statistically significantly higher than those residents who were far away from landfill sites with respect to wheezing (*p* < 0.0001), frequent sneezing (*p* < 0.0001), unpleasant odour (*p* < 0.0001), fever (*p* < 0.0001) and skin rashes (*p* < 0.0001) (Table 5).

## Discussion

This study enrolled household heads that share similar characteristics and were comparable in many socio-demographic characteristics except for marital status and proximity of their workplace to dumpsite and length of stay in residential location. This is important to ensure that respondents in the two groups were comparable and avoid groups that were different from each other so that any difference can be ascribed to distance from dumpsite and study results externally valid and generalisable.

In this study, respondents who work near dumpsite were statistically significantly more in Igando (79.6%) compared to Badagry (15.5%). This can be attributed to the fact that Igando is closer to landfill site than Badagry and workers prefer to live near their place of work.

The fact that the duration of stay in residential location was statistically significantly lower amongst Igando respondents compared to Badagry respondents and this could be because of the negative health and environmental conditions experienced by Igando residents who live close to landfill site. Hence, Igando residents tend to relocate to a more conducive residential area as soon as possible.

The findings of high mean knowledge score in respiratory and skin disorders associated with solid waste dump sites amongst the respondents, and the findings that majority of the respondents in the two groups had good knowledge of respiratory and skin conditions associated with solid waste dump sites are similar to the findings of a study carried out in Swaziland where majority (74%) of the respondents expressed good knowledge of the potential health and environmental effects that landfill could have on their community.^11^ The current findings also seem to be consistent with other research which found that amongst the residents of Aba, Umuaiha and Owerri in Nigeria who live close to waste dump sites, 61.1% of the respondents were enlightened about the possible health impact of the waste in their neighbourhood.^12^ Also, the research indicated that air pollution was rated highest (2.2 out of 3.0) because of the effect of solid waste dump sites on residents by the respondents.^12^

The high level of knowledge in our study may be because of the fact that the majority of the respondents in both locations (90.6% of Igando respondents and 84.4% of Badagry respondents) had at least secondary school education; however, there was no statistically significant relationship between any socio-demographic variable and knowledge.

The fact that the mean knowledge score in respiratory and skin disorders associated with solid waste dump sites of respondents close to landfill sites (82.53 ± 20.60) was statistically significantly higher than those of respondents far from landfill sites (71.84 ± 20.57) (*p* = 0.0003) can be attributed to the fact that those who are close to landfill sites are familiar with the health and environmental impact of closeness to landfill sites.

Another important finding in this study was that more of the respondents close to landfill sites reported respiratory and skin disorders than respondents far from landfill site. The majority of the respondents in Igando reported frequent sneezing, skin rashes, fever and cough which implies worsening of their health status than respondents living in Badagry. A possible explanation for this might be the close proximity (within 200 m) of the Igando respondents to the landfill site, whilst the Badagry respondents are about 5 km away from landfill site. However, the analysis of the result showed that respiratory and skin disorder experiences of respondents close to landfill sites were statistically significantly higher than those of the residents far from landfill sites with respect to wheezing (*p <* 0.0001), frequent sneezing (*p <* 0.0001), unpleasant odour (*p <* 0.0001), fever (*p <* 0.0001) and skin rashes (*p <* 0.0001),whereas there was no statistically significant relationship (*p* = 0.06) found between the frequency of cough reported and the location of the respondents.

Other skin and respiratory disorders experienced by the Igando and Badagry respondents were also found to be present amongst study subjects of a research work carried out in India.^13^ This study established that about 89% of a landfill workers exhibited single or multiple respiratory symptoms in contrast to 57% of the controls. Upper respiratory symptoms were found in 72% of the landfill workers compared to 37% of the controls.^13^ Similarly, lower respiratory symptoms were more prevalent in landfill workers (77%) than in controls (43%).^13^ Common upper respiratory symptoms in landfill workers were frequent sneezing, common cold and fever, running or stuffy nose and sinusitis. Chest discomfort or pain, breathlessness on exertion, cough with phlegm, dry cough and wheezing were the common lower respiratory symptoms found in the landfill workers.^13^

This study is also slightly similar to a referenced research done in Japan which focuses on the relationship between the prevalence of allergic disorders and the distance of schools from the incineration plant.^14^ The research found that schools closer to the nearest municipal waste incineration plants were found to have increased prevalence of respiratory disorders but there was no evident relationship between distance of schools from incineration plants and prevalence of atopic dermatitis.^14^

It is somewhat surprising that only 45% of Igando respondents reported common cold as a symptom of a respiratory disorder whilst 50% of Badagry respondents reported it. The findings of the result also showed that there was no statistically significant difference (*p* = 0.49) in the frequency of common cold reported with respect to the location of respondents. A possible explanation for this finding may be the inability of the respondents to associate waste dump sites as a risk factor of common cold. Another possible explanation may be their inability to recall past experiences.

This combination of findings in this study provide some support for conceptual premise that dumpsites near residential areas contribute to the skin and respiratory disorders experienced by residents.

## Limitations

Information on the living conditions, household air pollution, crowding, exposure to second hand smoke could not be obtained from the respondents; however, these could be a limitation on the findings of this study. This calls for the need for further research to investigate the possible effect of the above factors.

## Conclusion

The study revealed that living at the proximity close to the landfill site has great effect on the respiratory and skin disorder of people as noticed amongst the people living around Solous landfill site unlike Badagry people who live far away from landfill but are having cold because of their proximity to the sea.

Solid waste dump sites should be located far away from residential areas as these contribute unpleasant odour and sight to the residences. Effective campaign and health education should be adopted by the state government to enlighten the populace on danger associated with living near waste dump sites. The presently situated waste sites near residential areas should be properly managed to minimise the negative health impacts on the nearby residents.
